# An in situ inferior vena cava ligation-stenosis model to study thrombin generation rates with flow

**DOI:** 10.1186/s12959-022-00391-1

**Published:** 2022-05-25

**Authors:** Wei Yin, Andrew Dimatteo, Andrew Kumpfbeck, Stephen Leung, Marina Fandaros, Bryan Musmacker, David A. Rubenstein, Mary D. Frame

**Affiliations:** Department of Biomedical Engineering, Stony Brook University, Bioengineering Building, Room 109, Stony Brook, NY 11794 USA

**Keywords:** Inferior vena cava, Ligation, Platelet, Thrombin generation

## Abstract

**Background:**

Blood flow-induced shear stress affects platelet participation in coagulation and thrombin generation. We aimed to develop an *in vivo* model to characterize thrombin generation rates under flow.

**Methods:**

An in situ inferior vena cava (IVC) ligation-stenosis model was established using C57BL/6 mice. Wild type C57BL/6 mice were fed normal chow diet for two weeks before experiments. On the day of experiments, mice were anesthetized, followed by an incision through the abdominal skin to expose the IVC, which was then ligated (followed by reperfusion through a stenosis for up to 2 h). IVC blood flow rate was monitored using a Transonic ultrasound flow meter. In sham animals, the IVC was exposed following the same procedure, but no ligation was applied. Thrombin generation following IVC ligation was estimated by measuring mouse plasma prothrombin fragment 1–2 concentration. Mouse plasma factor Va concentration was measured using phospholipids and a modified prothrombinase assay. Blood vessel histomorphology, vascular wall ICAM-1, von Willebrand Factor, tissue factor, and PECAM-1 expression were measured using immunofluorescence microscopy.

**Results:**

IVC blood flow rate increased immediately following ligation and stenosis formation. Sizable clots formed in mouse IVC following ligation and stenosis formation. Both plasma factor Va and prothrombin fragment 1–2 concentration reduced significantly following IVC ligation/stenosis, while no changes were observed with ICAM-1, von Willebrand Factor, tissue factor and PECAM-1 expression.

**Conclusion:**

Clot formation was successful. However, the prothrombin-thrombin conversion rate constant *in vivo* cannot be determined as local thrombin and FVa concentration (at the injury site) cannot be accurately measured. Modification to the animal model is needed to further the investigation.

## Background

Platelets play important roles in thrombosis and atherosclerosis. It is well established that altered shear stress induced by disturbed blood flow can cause significant changes in platelet activity [1–4]. Activated platelets proceed through many morphological changes, including phospholipid flipping from the inner cell membrane to the outer cell membrane and the release of many pro-coagulant factors from the α-granules. By providing a negatively charged cellular surface (through phosphatidylserine expression), activated platelets can support coagulation cascade complex formation, enabling many of the subsequent enzymatic reaction to occur rapidly [5]. Activated platelets also release activated factor V (FVa) locally, accelerating prothrombin to thrombin conversion [6]. Vascular wall endothelial cells (EC) interact with platelets and can contribute to thrombin generation as well. Pathological shear stress can cause EC activation and sub-endothelium exposure [7], making the vascular wall pro-thrombotic. Enhanced tissue factor and von Willebrand factor (vWF) expression on the injured blood vessel wall is likely to promote platelet adhesion and initiate the extrinsic pathway coagulation.

Quantifying thrombin generation rates (and kinetics) is important. When small amounts of thrombin are generated slowly during the initial stage of coagulation, only loosely-woven clots are formed; in contrast, a large quantity of thrombin generated rapidly during the propagation phase of coagulation produces stable clots that are more resistant to fibrinolysis [8]. A better understanding of thrombin generation rates/kinetics under dynamic blood flow can help clinicians and researchers to better characterize specific phases in thrombin production and clot formation [9], and identify factors that regulate thrombosis and other clotting disorders [10]. To mathematically describe platelet thrombin generation, multiple kinetics models have been developed. The reaction of prothrombin conversion to thrombin by the prothrombinase complex can be described with an overall “prothrombinase” rate constant or multiple rate constants, describing the individual reactions and interactions between factor Xa, factor V/Va, and/or phosphatidylserine. We previously reported that under flow conditions, thrombin generation rate was not only dependent on shear stress conditions (i.e., magnitude, pattern, exposure time, etc.), but also the immediate availability of coagulation proteins [11, 12]. Our results demonstrated that the overall rate constant for prothrombin-thrombin conversion was not a “constant” when considering the complex conditions observed *in vivo*, but an ever-changing dynamic parameter.

Numerous *in vitro* and *ex vivo* models have been developed to study thrombin generation [11, 13, 14]. *In vivo* models are also widely used to study thrombus formation under various conditions [15]. Mechanical injury introduced by vessel transection, pinching, vessel occlusion, endothelial denudation, and vascular wall puncturing, etc. [16] in micro-vessels (such as the mesenteric arterioles) or larger vessels (such as the inferior vena cava) can all lead to rapid thrombus formation. Using those models, platelet adhesion, accumulation and interaction with leukocytes and vascular wall endothelial cells were studied. However, due to the complex *in vivo* environment, few have directly used animal models to determine rate constants of coagulation kinetics (especially prothrombin-thrombin conversion rate constant), to validate the kinetic constants obtained from *in vitro* or numerical models. In the present study, an in situ mouse inferior vena cava ligation/stenosis model was established to explore if the prothrombin-thrombin conversion rate constant can be determined under complex *in vivo* conditions, and how it is compared to those previously obtained from numerical and *in vitro* models.

## Methods

### The inferior vena cava ligation-stenosis model

An inferior vena cava (IVC) ligation-stenosis model was established using C57BL/6 mice. This model was originally developed to study acute or chronic deep vein thrombosis [17, 18]. Wild type C57BL/6 mice [10-12 weeks, male] were fed with normal chow diet. The demographic information of the mice is listed in Table 1. On the day of experiments, mice anesthesia was induced with 4–5% isoflurane (induction box) and then maintained on isoflurane (1–2%) using nose cone delivery. The ventral abdomen was cleaned and shaved, followed by an incision through the abdominal skin. The intestines were gently pulled out to expose the IVC. A 600 µm (O.D., 300 µm I.D.) PTFE tubing was placed along the IVC, and 000 size silk suture was used to tie the tubing within the IVC. The ligature was tied tightly with a single and then double tie (i.e., the locking tie), making it initially a full blood flow blockage. This full blood flow blockage lasted for approximately 5–10 s before the PTFE tubing was pulled loose, leaving the suture ligature. This allowed blood flow to resume in the IVC [19]. Blood flow passing through the stenosis was confirmed by ultrasound. The ligature was left in place for 1 or 2 h for thrombus to form [20]. In sham animals, the IVC was exposed following the same dissection procedure, but no ligation was applied. Local blood flow rate within the IVC was monitored using a Transonic flow meter (TS420 Perivascular Flow Module, Transonic Systems Inc., Ithaca, NY). Blood flow readings were taken before ligation and every 5 min during the first hour, and every 15 min during the second hour. Before IVC ligation, 25 µL blood was collected via toe clip. At the end of the experiment (1 h or 2 h), 200—300 μL of blood was drawn by cardiac puncture into EDTA washed syringes.

**Table 1:** Baseline demographics of the mice used in the study

Average of weight (g)	24.58 (3.19)
Average of age (days)	87.9 (22.5)
Average of hematocrit (%)	44.9 (11.7)

Entries are Mean (Standard Deviation)

### Platelet activation, thrombin generation and factor Va concentration

Whole mouse blood (with 3.8% sodium citrate) was centrifuged at 100 × *g* for 7 min to obtain platelet rich plasma (PRP). PRP was incubated with FITC-conjugated CD62P antibody at room temperature for 30 min, followed by platelet activation measurement using flow cytometry (Accuri C6, BD).

To determine the amount of thrombin generated due to IVC ligation/stenosis, PRP was further centrifuged (1000 × *g,* 5 min) to obtain platelet poor plasma (PPP). A commercial ELISA kit (MyBioSource Inc., San Diego, CA) was used to measure mouse plasma prothrombin fragment 1–2 concentration [21]. Prothrombin fragment 1–2 are cleaved from prothrombin by factor Xa during thrombin formation [22], therefore, their plasma concentration is often used as an indicator of plasma thrombin concentration, for the diagnosis of thrombosis and other clotting disorders [23].

To measure plasma factor Va concentration, mouse PPP was incubated with phospholipid membranes (30% phosphatidylserine and 70% phosphatidylcholine, Sigma Aldrich, St. Louis, MO) [24, 25], factor II (1.41 μM, Enzyme Research Laboratories), factor Xa (1 nM, Enzyme Research Laboratories) and Ca^2+^ (5 mM) at 37 °C for 10 min. Thrombin generation was measured using Chromozym-Th (Roche, absorbance at 405 nm) and factor Va concentration was calculated using a standard curve.

### Endothelial protein expression

Following euthanasia, mouse IVC was collected, fixed with 4% paraformaldehyde (overnight), frozen in Tissue-Tek® OCT medium and sliced. Blood vessel wall histomorphology and integrity was examined under a light microscope. Blood vessel wall ICAM-1, PECAM-1, tissue factor, and von Willebrand Factor (vWF) expression was measured using immunofluorescence microscopy (rabbit anti mouse ICAM-1, PECAM-1 and vWF antibodies were purchased from Abcam, and used at 1 µg/mL). For experimental control, no primary antibody but PBS was added to the sliced blood vessel samples. Secondary antibody was then added (Alexa Fluor 488-conjugated goat anti rabbit antibody, obtained from Abcam and used at 1 µg/mL), followed by immunofluorescence microscopy, to determine the fluorescence intensity of bound antibody. Image analysis was conducted using the *Image J* software. All intensity values were normalized to that of matching negative control.

### Statistical analysis

Student’s *t*-test was used to compare the flow rate, plasma factor Va concentration, plasma prothrombin fragment 1–2 concentration, and blood vessel wall coagulant protein (vWF, ICAM-1, tissue factor and PECAM-1) expression between sham and ligated (stenosis) mice. A *P*-value of 0.05 was considered significant.

## Results

### IVC flow rate

**Fig. 1:**
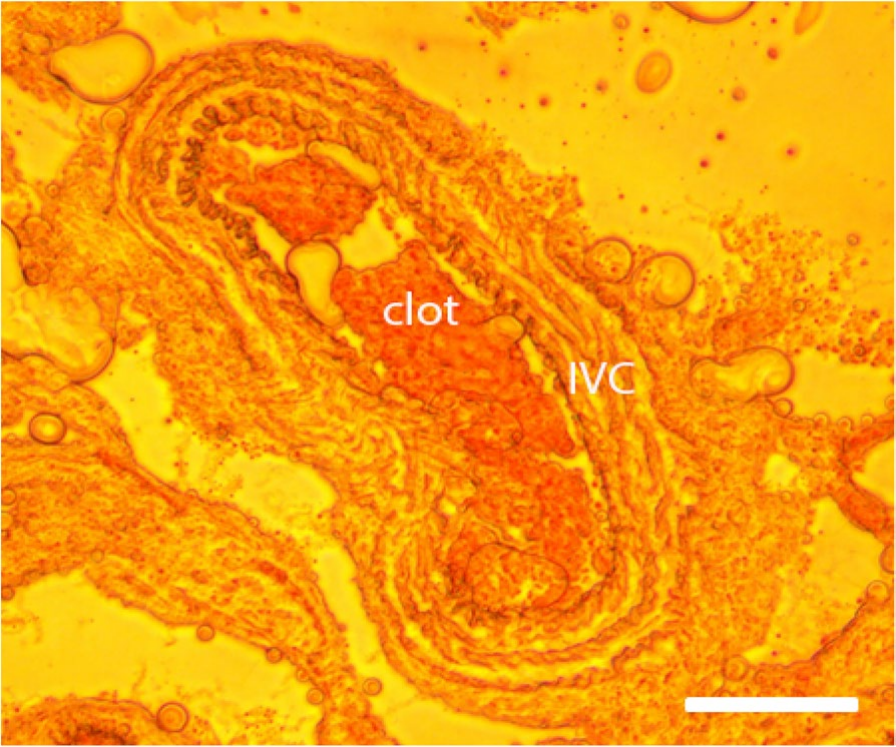
A representative image of a ligated/stenosed inferior vena cava (IVC). The nominal long axis is approximately 400 µm, and the vessel appears constricted. One hour after ligation/stenosis formation, clots were visible within the blood vessel. Scale bar represents 100 µm

Histomorphology examination of the ligated/stenosed IVC under a light microscope indicated that blood clots formed in the blood vessel within 1 h following ligation/stenosis formation, demonstrating the ligating procedure was effective. Figure 1 depicts a typical ligated IVC (1 h) with visible clots. No clot was observed in IVC collected from sham mice.

**Fig. 2:**
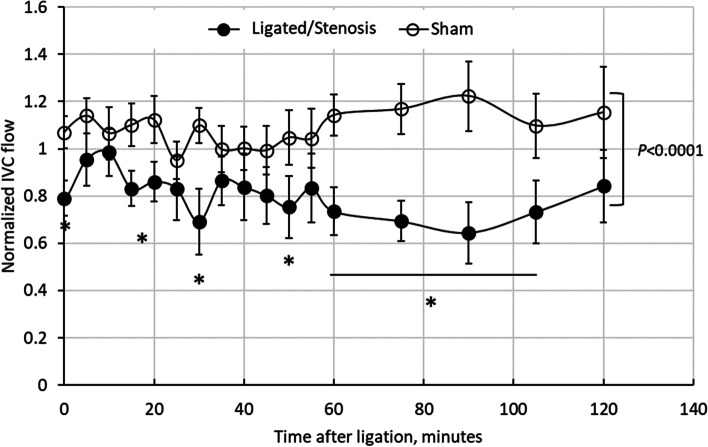
Normalized flow rate in sham and ligated/stenosis mice. All numbers were normalized to the baseline flow rate values before ligation or sham equivalent. Data is presented as mean ± standard error (*n* = 8–15). *differs from sham at the same time point

The normalized flow rates (to baseline level) in sham and ligated mice are depicted in Fig. 2. No noticeable difference in flow rate was observed in sham and ligated animals immediately before (t < 0) the operation (*P* > 0.05). Student’s *t*-test indicated that the difference in normalized flow rates between all sham and ligated/stenosis mice independent of time after ligation was significant (sham: 1.07 + 0.025, *n* = 200 vs ligated: 0.81 + 0.025, *n* = 204, *P* < 0.0001). In ligated/stenosis mice, following IVC ligation, the flow rate instantly (t = 0) dropped by more than 20%. Within the first 30 min after ligation, flow rate varied between 70 and 100%. By 60 min, flow rate was reduced to about 73% in ligated/stenosis mice, and maintained so (64–84%) through the second hour. In contrast, flow rate in sham animals had smaller variations during the first 60 min (95%—114%), and gradually increased during the second hour (110–122%).

### Platelet activation, factor Va release and thrombin generation

**Fig. 3:**
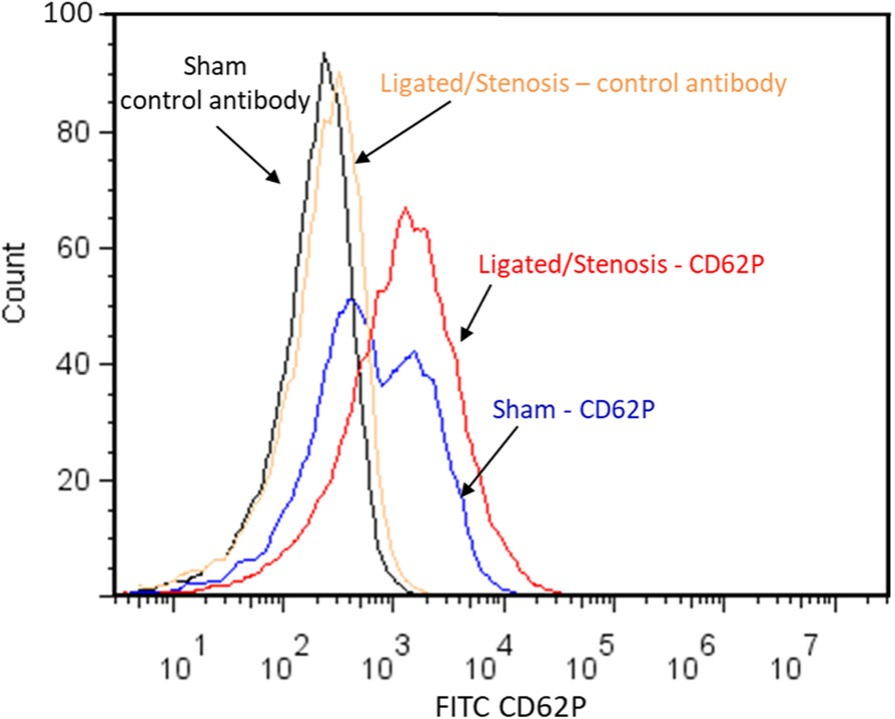
Representative fluorescence intensity histograms of CD62P expression on platelets from sham and ligated/stenosis mice. CD62P-positive cell population shifted to the right following ligation, indicating ligation caused platelet activation. Non-specific antibody binding was measured using a rat anti-mouse IgG isotype control antibody

To evaluate the activation state of platelets, whole blood collected at the end of experiments was centrifuged. PRP was collected and incubated with FITC-conjugated CD62P (P-selectin) antibody, followed by flow cytometry immediately. Non-specific antibody binding was detected using a rat anti-mouse IgG isotype control antibody. The representative fluorescence intensity histograms of CD62P expression on platelets from sham and ligated/stenosis animals are shown in Fig. 3. Following ligation/stenosis formation (1 h), the CD62P-positive cell population shifted to the right, indicating platelet activation.

**Fig. 4:**
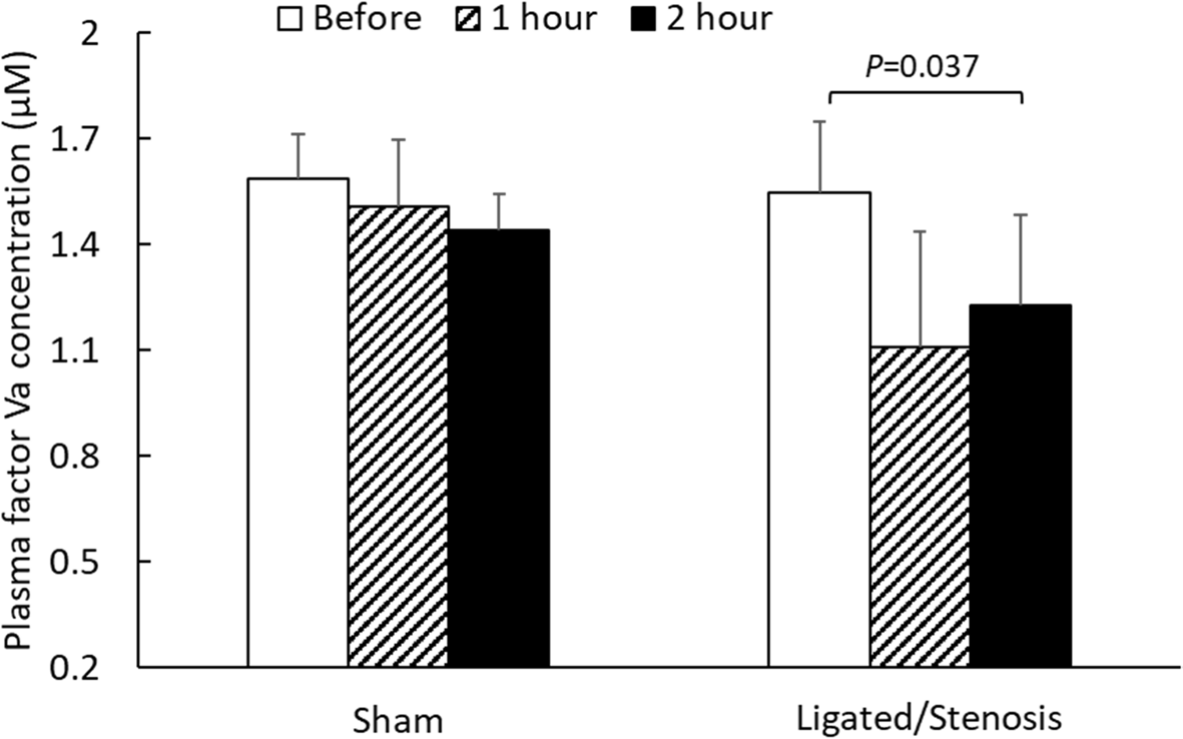
Plasma factor Va concentration in sham and ligated/stenosis mice. Data is presented as mean + standard error (*n* = 4–7). Significant difference (*P* < 0.05) is marked

Plasma Factor Va concentration in sham and ligated/stenosis mice was measured using the phospholipid-prothrombinase assay, and the results are shown in Fig. 4. In sham animals, Factor Va concentration did not change much through the 2 h experiment. Immediately before the experiment, plasma factor Va concentration was 1.58 ± 0.13 µM (mean ± standard error, *n* = 7); at 1 h, plasma factor Va concentration was 1.51 ± 0.19 µM (*n* = 6), and at 2 h, plasma factor Va concentration was 1.44 ± 0.10 µM (*n* = 4). For the ligated/stenosis mice, however, significant changes in Factor Va concentration was observed. Immediately before the operation, plasma factor Va concentration was 1.55 ± 0.20 µM (*n* = 7), which decreased to 1.11 ± 0.33 µM (*n* = 4) at 1 h, and 1.23 ± 0.26 µM (*n* = 4) at 2 h. Paired Student’s *t*-tests were used to compare plasma factor Va concentration in the same mouse before and after (1 h or 2 h) the operation. The results demonstrated that 2-h ligation/stenosis caused a significant decrease (*P* = 0.037) in mouse plasma factor Va concentration. Noticeable decreases were observed in mice ligated for 1 h, however, due to large variance in the collected data, no statistically significant decrease (*P* = 0.12) was detected.

**Fig. 5:**
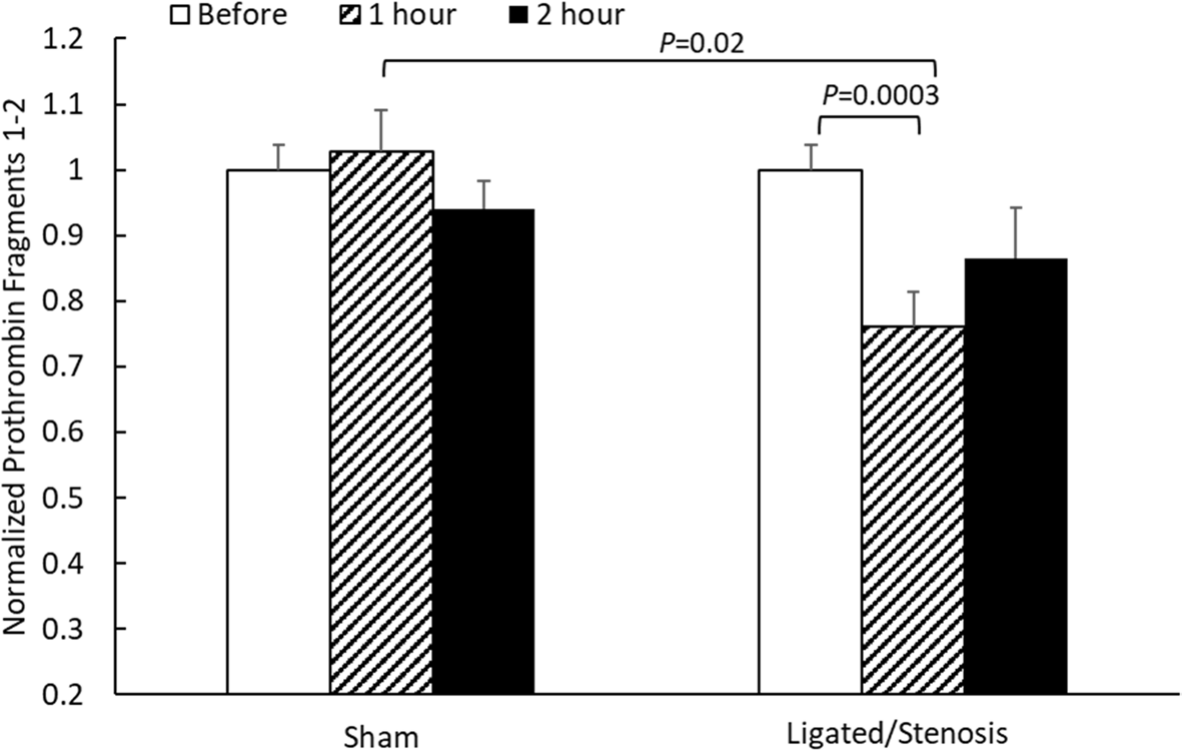
Normalized plasma prothrombin fragment 1–2 concentration in sham and ligated/stenosis mice. All data is presented as mean + standard error (*n* = 4–9). Significant difference is marked. Average plasma prothrombin fragment 1–2 concentration before operation (sham and ligation, *n* = 17) was 1.27 ± 0.05 µg/mL

A similar trend was observed with the plasma concentration of prothrombin fragment 1–2, which were released from prothrombin during thrombin formation. Figure 5 depicts normalized prothrombin fragment 1–2 concentration in mouse plasma before and after ligation (or sham equivalent). Plasma prothrombin fragment 1–2 concentration in each individual mouse before the operation was used to normalize the data obtained from the same mouse. On average, plasma prothrombin fragment 1–2 concentration before the operation was 1.27 ± 0.05 µg/mL (mean ± standard error, *n* = 17). For the sham animals, 1 h after the operation, plasma prothrombin fragment 1–2 concentration remained about the same (normalized value ± standard error: 1.03 ± 0.06, *n* = 7); and at 2 h, it dropped slightly to 0.94 ± 0.04, *n* = 4). No statistically significant difference was detected between the conditions. For the ligated/stenosis mice, however, significant difference (*P* < 0.05 by paired Student’s *t*-test) was noticed. 1-h ligation/stenosis reduced the normalized plasma prothrombin fragment 1–2 concentration to 0.76 ± 0.05 (*n* = 4, *P* = 0.0003), and 2-h ligation reduced that to 0.86 ± 0.08 (*n* = 4). When sham mice and ligated/stenosis mice were compared, sham mice at 1 h also had a significantly higher prothrombin fragment 1–2 concentration compared to ligated/stenosis mice at 1 h (*P* = 0.02 by Student’s *t*-test).

### Endothelial cell adhesion and coagulant protein expression

**Fig. 6:**
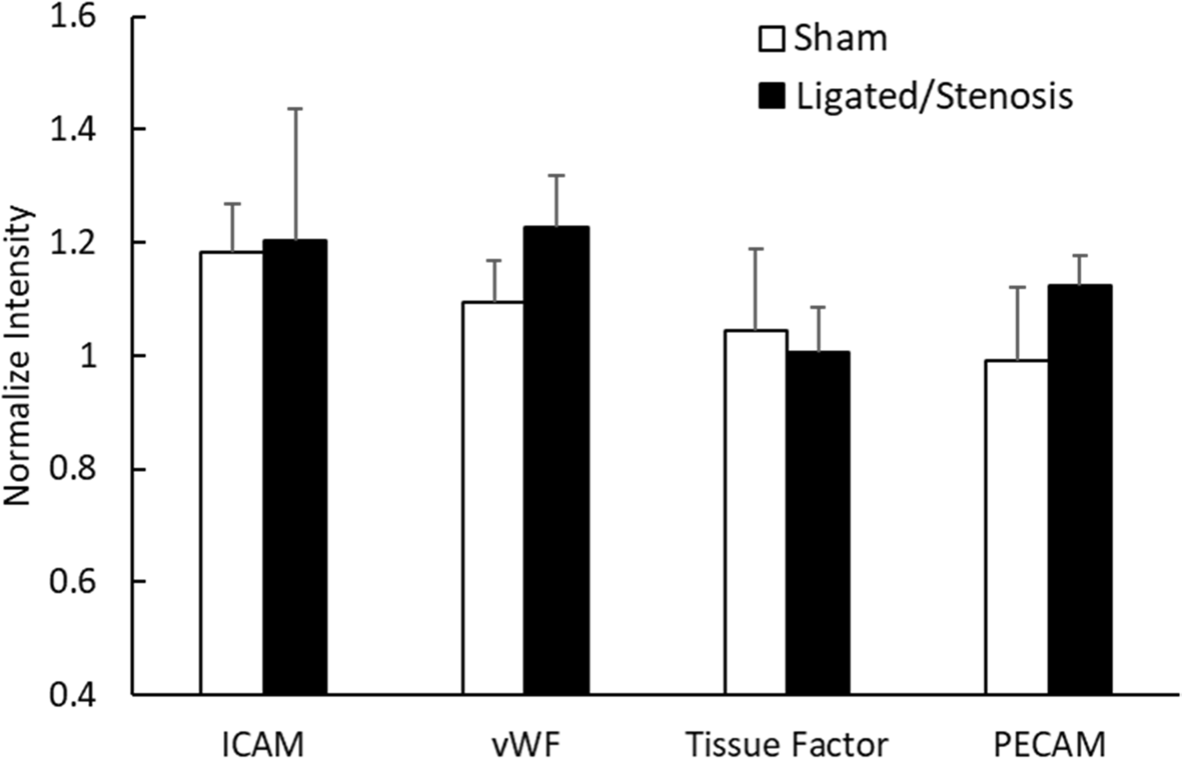
ICAM-1, von Willebrand Factor (vWF), tissue factor (TF) an PECAM-1 expression on the blood vessel wall 2 h after ligation, measured using immunofluorescence microscopy. Data is presented as mean + standard error, *n* = 5–7. No significant difference was detected

Mouse IVC blood vessel wall ICAM-1, von Willebrand Factor (vWF), tissue factor and PECAM-1 expression was measured using immunofluorescence microscopy. Image analysis was conducted using the *Image J* software. All intensity values were normalized to that of the negative control. As depicted in Fig. 6, following 2-h of IVC ligation/stenosis, slight increases were observed in vWF (13%, *n* = 4–7) and PECAM-1 (13%, *n* = 4–7) expression in ligated/stenosis mice. However, no statistical significance was detected. Changes in ICAM-1 and tissue factor expression was minimal.

## Discussion

Thrombin generation is dynamic and dependent on multiple factors, including blood flow, shear rate, coagulation factors, and substrates. This study aimed to use a mouse inferior vena cava ligation (followed by stenosis) model to quantify thrombin generation rate (and calculate thrombin generation rate constant) under flow conditions *in vivo*. Compared to our previously reported *in vitro* model [11], a lot more factors were involved (in the current *in vivo* model) in thrombin generation, including circulating blood flow (which can dilute local coagulation protein concentration and carry away coagulation products), plasma proteins (which can promote or inhibit coagulation), and endothelial cell surface proteins. All these factors imposed significant challenges in measuring local factor Va and thrombin concentration (or prothrombin fragment 1–2 concentration) at the site of ligation/stenosis.

The inferior vena cava ligation model and stenosis model are well-adapted models [26–28]. In this study, a full blood flow blockage was introduced using a PTFE tubing and silk ligature, following standard ligation procedures. This full blood flow blockage lasted for 5–10 s before the PTFE tubing was pulled away, allowing blood flow to resume (reperfusion) through a stenosis. As demonstrated in Fig. 2, the flow rate in interior vena cava decreased following ligation (through the stenosed region), and remained low (compared to the sham animals) throughout the 1–2 h period following ligation. Clots were observed in the blood vessel after 1-h following ligation (as shown in Fig. 1). It was reported by others that the ligation procedure could induce platelet activation and adhesion [29] at the injury site, which was in agreement with our observation that following ligation/stenosis formation, platelet surface P selectin expression increased. No significant changes were observed in ICAM-1, vWF, tissue factor and PECAM-1 expression on vascular endothelial cells (Fig. 6). This may have resulted from the relatively short duration of our experiment (2 h), and may suggest that the observed changes in platelets and thrombin generation was not due to endothelial cell activation.

With platelet activation and clot formation, we had expected to see increased plasma factor Va concentration and thrombin generation. However, decreased plasma factor Va concentration and prothrombin fragment 1–2 concentration were observed in ligated/stenosis mice one or two hours following ligation, different from that observed in our previously reported *in vitro* model [11]. In literatures on inferior vena cava ligation/stenosis models, thrombosis and clot sizes were often used to evaluate stenosis severity [26, 30], while plasma factor Va (released by activated platelets) and thrombin concentrations were rarely reported. *In vivo*, factor Va is generated by thrombin cleavage of plasma factor V, and is required in prothrombinase complex formation [31]. Factor Va can be rapidly inactivated by activated protein C, which is catalyzed by thrombin bound to thrombomodulin on vascular endothelial cells [32]. Gale et al. reported that factor Va was quite unstable in plasma. Using an APTT (activated partial thromboplastin time) assay, they demonstrated that factor Va activity decreased by 75%-80% within 2 h in citrated normal human plasma [33]. In vivo, platelet-released factor Va plays a more important role (compared to plasma factor V) in binding to damaged endothelial cells and clotting when vascular injury occurs in larger vessels [34]. However, extra dosage of plasma factor V/Va can be cleared by the body rather quickly (plasma FV half-life was 124 min) [33]. Therefore, the observed decrease in factor Va concentration one to two hours following inferior vena cava ligation could have resulted from the combined effects of factor Va instability and plasma factor V/Va clearance.

Prothrombin fragment 1–2 is released during prothrombin activation to thrombin by the prothrombinase complex. Plasma prothrombin fragment 1–2 concentration has been used as a clinical biomarker for thrombosis. Multiple reports have demonstrated that decreased prothrombin fragment 1–2 level is associated with decreased thrombin generation [35], while increased prothrombin fragment 1–2 level indicates higher risk of thrombosis [36–38]. Prothrombin fragment 1–2 has been considered a reliable measure of thrombin generation due to its relatively long half-life (90 min), in comparison to that of thrombin (about 1 min) [39, 40]. A gradual decrease in prothrombin fragment 1–2 level in vivo was usually observed in deep vein thrombosis patients treated with anticoagulant [41]. However, in a total hip arthroplasty study by Borgen and Reikeras, a significant decrease in plasma prothrombin fragment 1–2 was observed one day after the surgery, which was explained by the combined effects from plasma anti- or pro-coagulant proteins, the dynamics of dilution, and blood loss [42]. In the current study, no anti-coagulants were given to the mice before or during the IVC ligation surgery, and no hemorrhaging was observed during the experiments. At the end of the experiment, blood was collected through cardiac puncture. Therefore, the observed decrease in prothrombin fragment 1–2 was likely resulted from the dynamics of dilution, or the activation of plasma coagulation inhibitors following a large clot formation. In vivo thrombin generation can be quantified in different ways, such as by measuring D-dimer (clotting degradation products), prothrombin fragment 1–2 (activation products), or thrombin-antithrombin (enzyme-inhibitor complex) [43]. In the current study, due to the small amount of blood collected from the mice, no alternative methods were used to verify if the measured prothrombin-fragment 1–2 concentration accurately represented the amount of thrombin generated. Therefore, other coagulation-associated products such as D-dimer or thrombin-antithrombin complex, may need to be measured to validate the estimated thrombin concentration (from prothrombin fragment 1–2).

To answer the question if this model can be used to quantify the overall prothrombin-thrombin conversion rate constant in vivo (with the presence of blood flow, endothelial cells, and plasma proteins), an extension of ternary kinetics could be used as described previously [11], assuming that the rate constant was only dependent on the initial prothrombin concentration, exogenous factor Va and available phosphatidylserine. It was assumed that calcium concentration and factor Xa concentration was constant and abundant and thus not rate-limiting. Prothrombin was available and the initial concentration was 109 µg/mL in mouse plasma [44]. We could also assume that under flow there was approximately 75 nM of phosphatidylserine (PS) available on platelet surface [11] to participate in coagulation. As shown in Eq. 1, prothrombin-thrombin conversion rate constant ($k$) can be estimated using the thrombin generation rate $\frac{d\left[IIa\right]}{dt}$, and the concentrations of prothrombin ($[II]$), factor Va ($[Va]$) and PS ($[PS]$).

1 $${v}_{0}=\frac{d[IIa]}{dt}=k\left[II\right]\left[Va\right]\left[PS\right]\to k=\frac{\frac{d[IIa]}{dt}}{\left[II\right]\left[Va\right][PS]}$$

Decreased plasma prothrombin fragment 1–2 was observed in this mouse IVC ligation model. If it indicated a decreased thrombin (IIa) concentration following ligation/stenosis, a negative and rather meaningless $k$ value would be obtained using Eq. 1. Thus, this model may not be suitable to estimate the prothrombin-thrombin conversion rate constant ($k$) *in vivo*, as local thrombin concentration (at the injury site) cannot be accurately measured.

Overall, this mouse IVC ligation-stenosis model helped to initiate our investigation in determining the prothrombin-thrombin conversion rate constant ($k$) *in vivo.* Clot formation was successful, indicating the coagulation cascade was activated and thrombin generated. Platelet activation and endothelial cell activation/inflammation could be measured using this model. However, due to plasma factor Va instability and clearance, as well as thrombin dilution during circulation, $[Va]$ and $\frac{d\left[IIa\right]}{dt}$ could not be accurately measured, making calculating the rate constant $k$ impossible. However, we speculate the *in vivo* rate constant $k$ is likely to be different from those obtained from *in vitro* assays (with or without flow).

It is very difficult to directly measure thrombin concentration/generation at the clotting site *in vivo*, due to blood flow diffusion and convection. In a study by Weslsh et al., a special thrombin sensitive antibody was developed and used to measure local thrombin concentration in real time in a murine model [45]. This antibody can bind to platelet GPIb (CD41) and contain a thrombin sensitive peptide, which can be cleaved by thrombin and generate a fluorescence signal. Using intravital microscopy, thrombin generation at the injury/clotting site can be monitored and quantified. In a report by Natatsuka et al., thrombin-activated microbubbles were used as the contrast agent to detect and quantify thrombin/clot formation (by acoustic activity) using ultrasound [46]. Therefore, special antibodies (or contrast agents) may need to be developed, and intravital microscopy (or other imaging modalities such as ultrasound) may need to be adopted, to more accurately quantify local thrombin concentration (at the clotting site). Thus, further investigation is needed and modifications are required to improve the current *in vivo* model.

## Data Availability

The datasets used and/or analyzed during the current study are available from the corresponding author on reasonable request.
